# Supplementary material for: Understanding voltage decay in lithium-excess layered cathode materials through oxygen-centred structural arrangement
Source: Nat Commun. 2018 Aug 16;9:3285. doi: 10.1038/s41467-018-05802-4 (PMC6095869; doi:10.1038/s41467-018-05802-4)
Supplement: Supplementary file 1 — Supplementary Information [file 41467_2018_5802_MOESM1_ESM.pdf]

Supplementary Information

Understanding voltage decay in lithium-excess layered cathode materials through oxygen-centred structural arrangement

by Myeong et al.

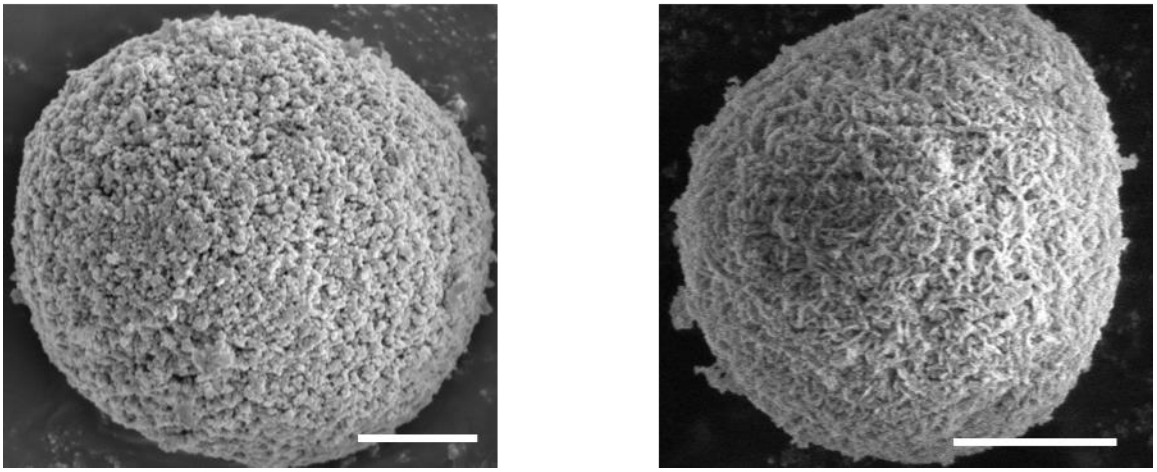

Supplementary Figure 1 | SEM image of samples. a) O-MNC and b) D-MNC. Scale bar denote 4 μm.

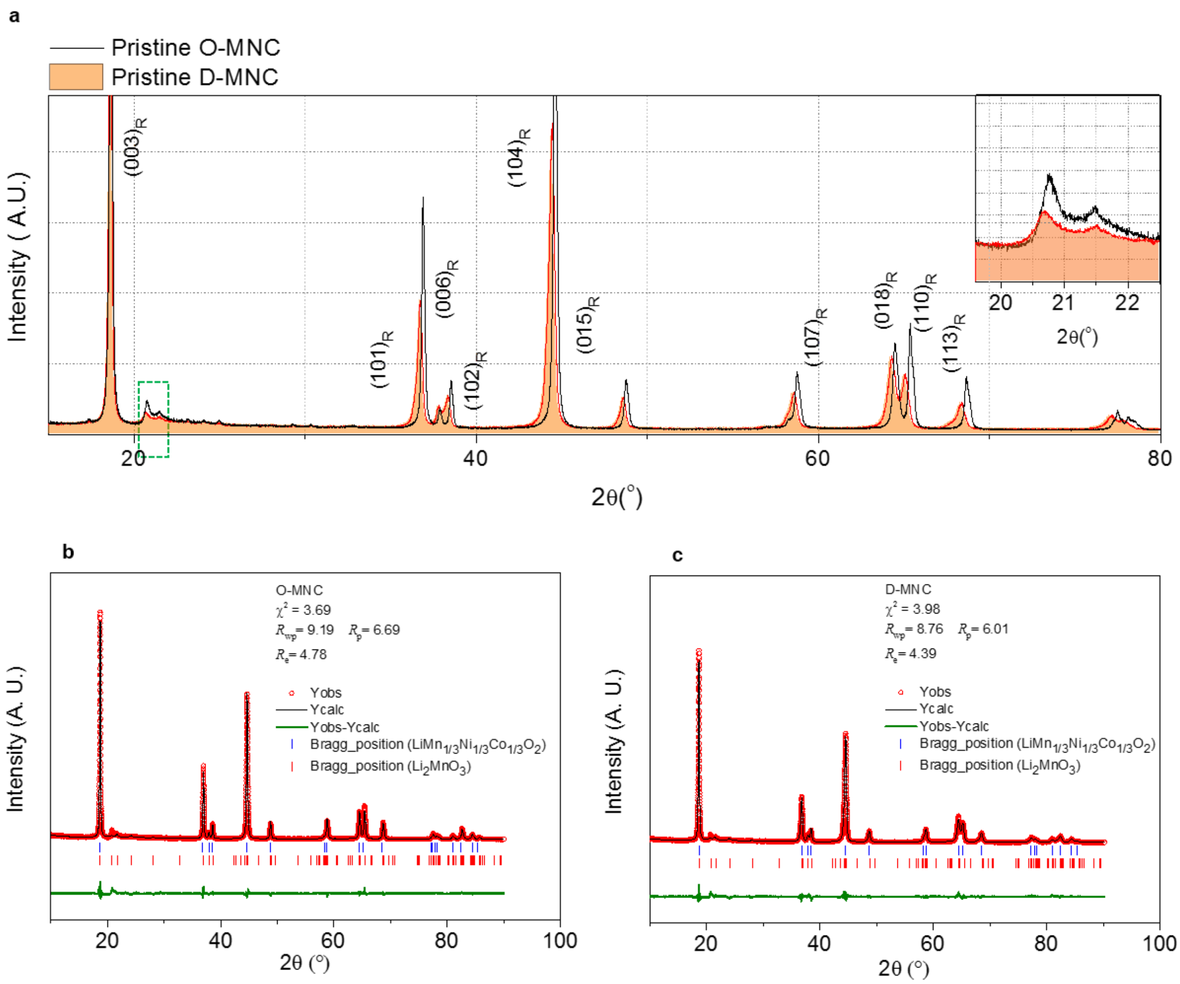

Supplementary Figure 2 | X-ray diffraction (XRD) pattern analysis of O-MNC and D-MNC

a) Comparison of XRD profiles of O-MNC and D-MNC. Rietveld refined XRD profiles of b) O-MNC and c) D-MNC. The reference phases (C2/m phase, R3m phase) and differences of fitness are also presented in figures. Interestingly, almost all XRD peak of D-MNC shows broad and blunt indicating the low crystalline structure compared to O-MNC. The green rectangle in **a** shows the reflections from Li-TM-TM ordering within the Li_2MnO_3-like structure. Overall XRD patterns of D-MNC show the broad Full Width at Half Maximum (FWHM) which well indicates the low crystallinity (cation-disordered layered phase and short-range ordered Li-TM-TM arrangement) of Li-excess 3dTM layered oxide material originating from cation disordering.

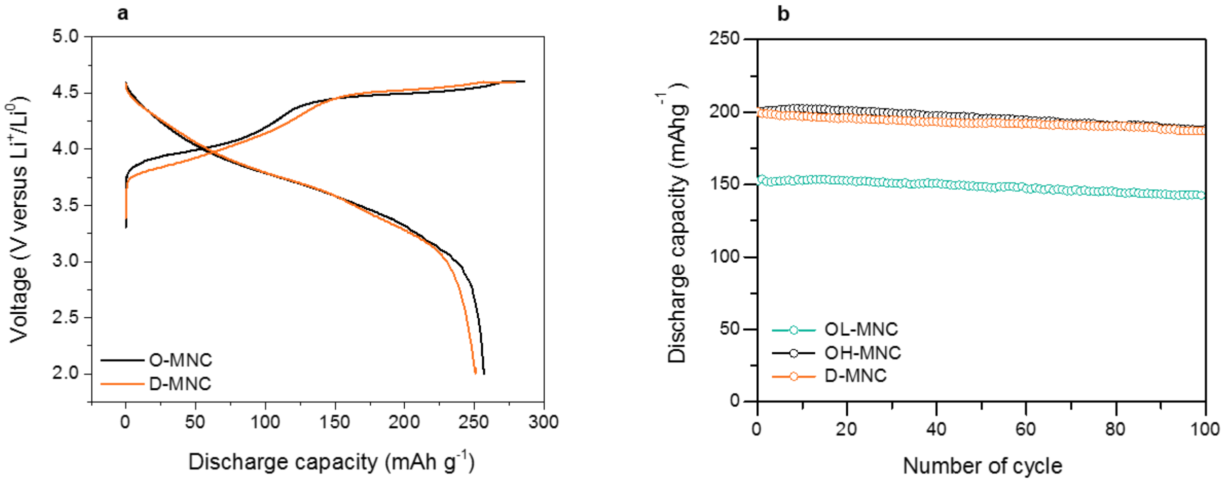

Supplementary Figure 3 | Electrochemical performance. a) Initial charge-discharge curves for O-MNC and D-MNC in the 2.00-4.60 V (versus Li-metal) potential region; 0.1 C-rate charge 0.1 C-rate discharge condition; b) cycle plot of OL-MNC, OH-MNC and D-MNC

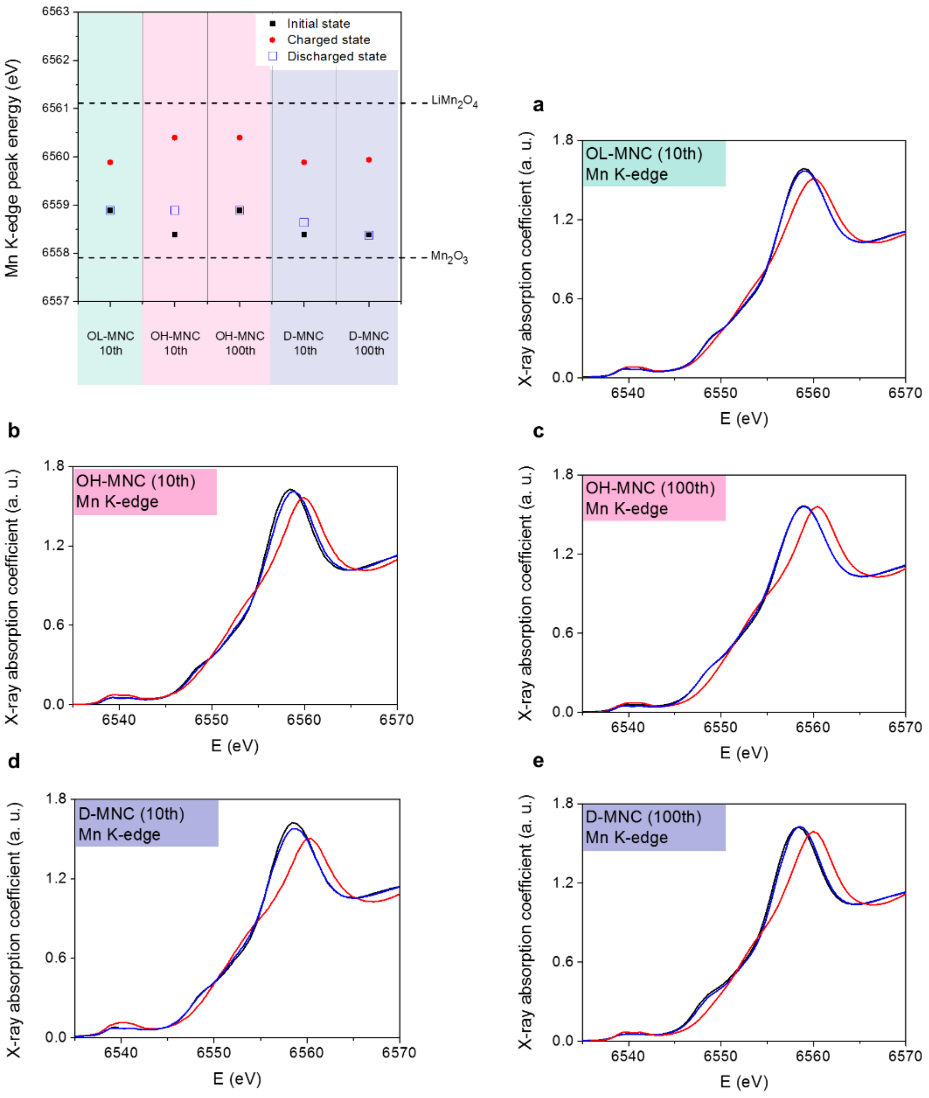

Supplementary Figure 4 | Oxidation state variation of Mn ion for OL-MNC, OH-MNC and D-MNC during cycling. Mn K-edge spectra of a) OL-MNC on 10th cycling b) OH-MNC on 10th cycling c) OH-MNC on 100th cycling d) D-MNC on 10th cycling e) D-MNC on 100th cycling.[1-3]

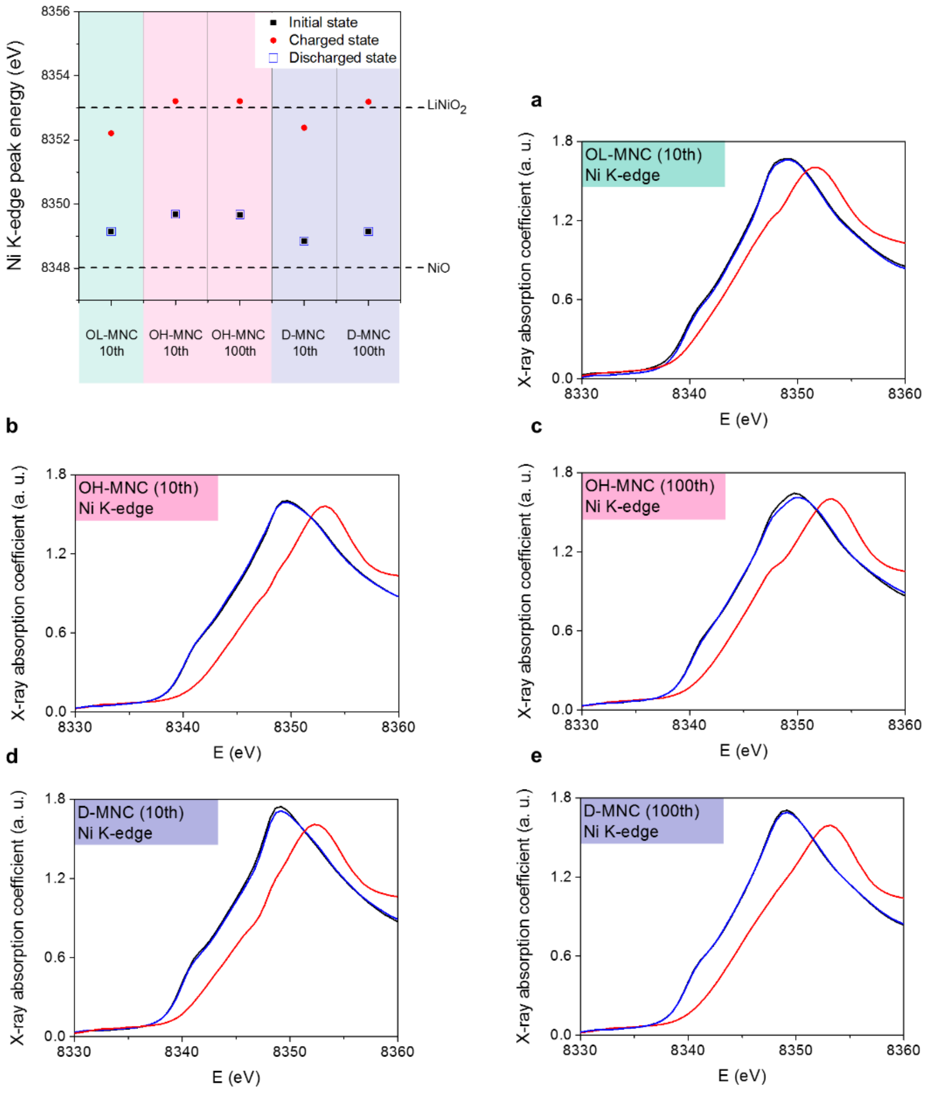

Supplementary Figure 5 | Oxidation state variation of Ni ion for OL-MNC, OH-MNC and D-MNC during cycling. Ni K-edge spectra of a) OL-MNC on 10th cycling b) OH-MNC on 10th cycling c) OH-MNC on 100th cycling d) D-MNC on 10th cycling e) D-MNC on 100th cycling.[1, 2]

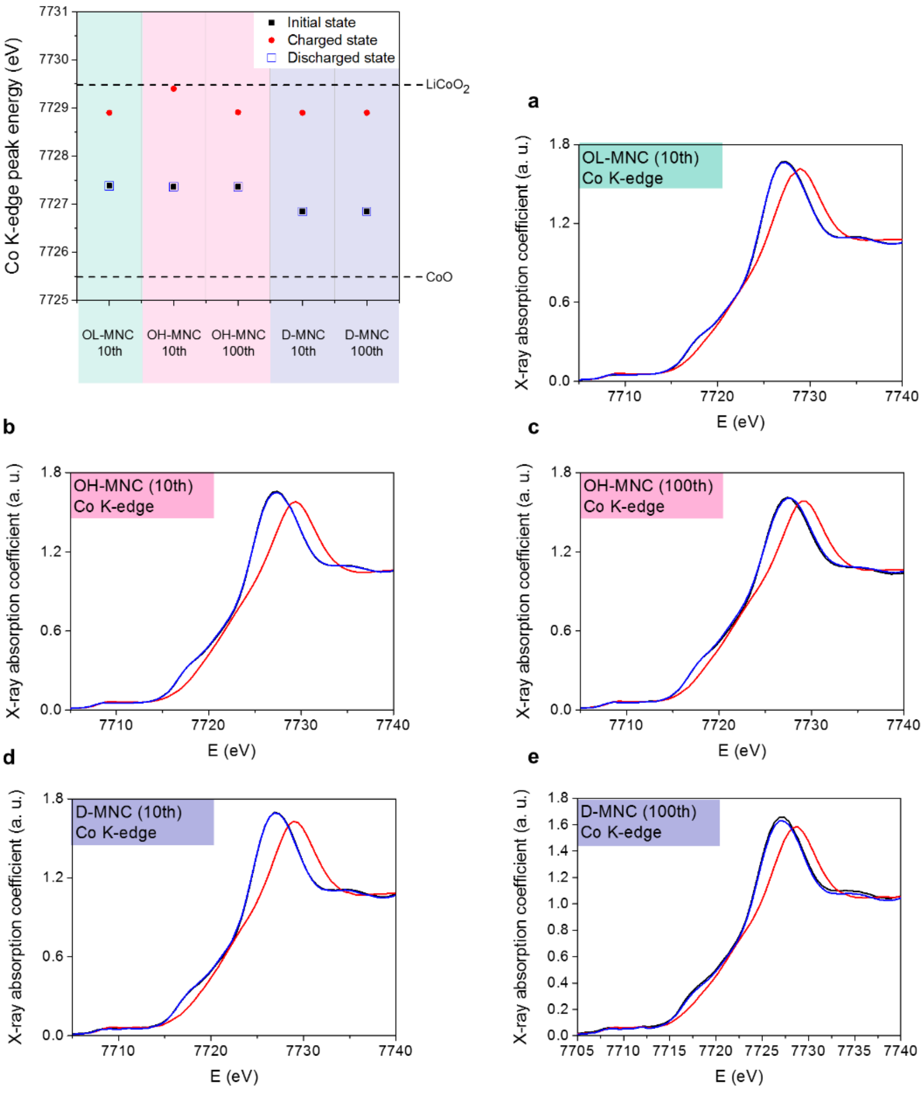

Supplementary Figure 6 | Oxidation state variation of Co ion for OL-MNC, OH-MNC and D-MNC during cycling. Co K-edge spectra of a) OL-MNC on 10th cycling b) OH-MNC on 10th cycling c) OH-MNC on 100th cycling d) D-MNC on 10th cycling e) D-MNC on 100th cycling.[1, 2]

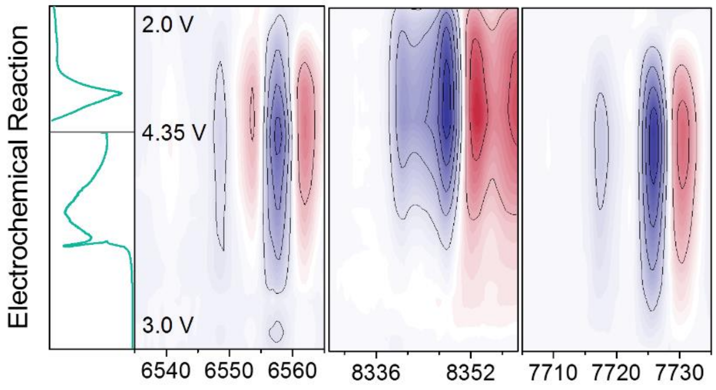

Supplementary Figure 7 | *Operando* XANES characterization of the OL-MNC during cycling. Normalized Mn, Ni and Co K-edge *operando* XANES spectra (2D contour plot) and voltage profiles of OL-MNC on 10th cycling.

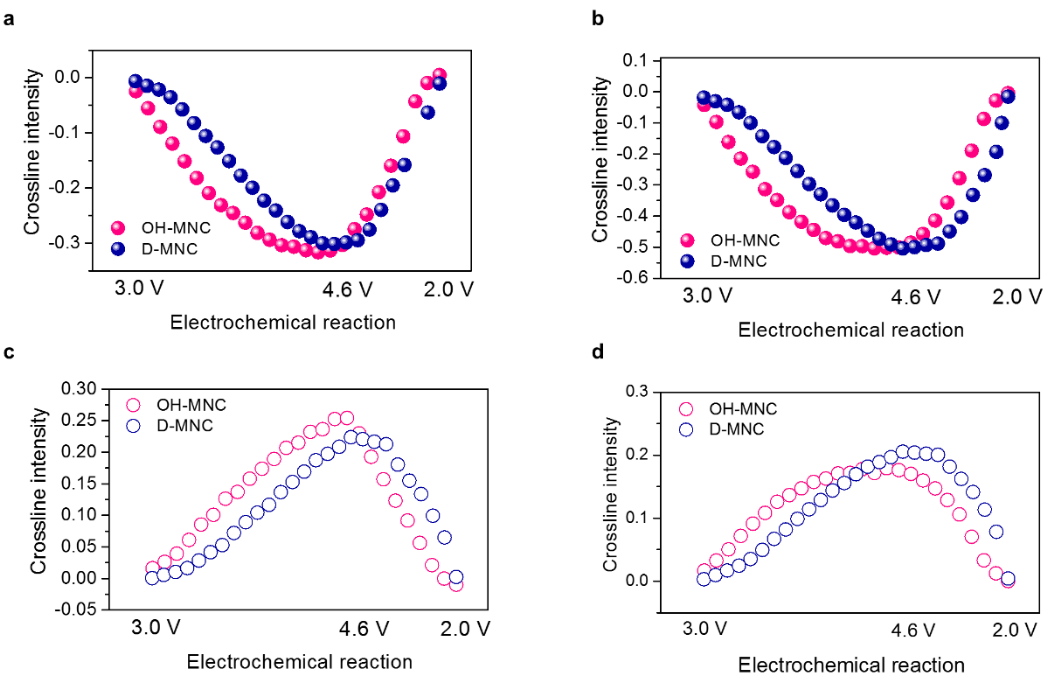

Supplementary Figure 8 | Crossline intensity of Ni K-edge *operando* XANES spectra during 10th cycle. Crossline intensity of a) peak A; b) peak B; c) peak; C d) peak D

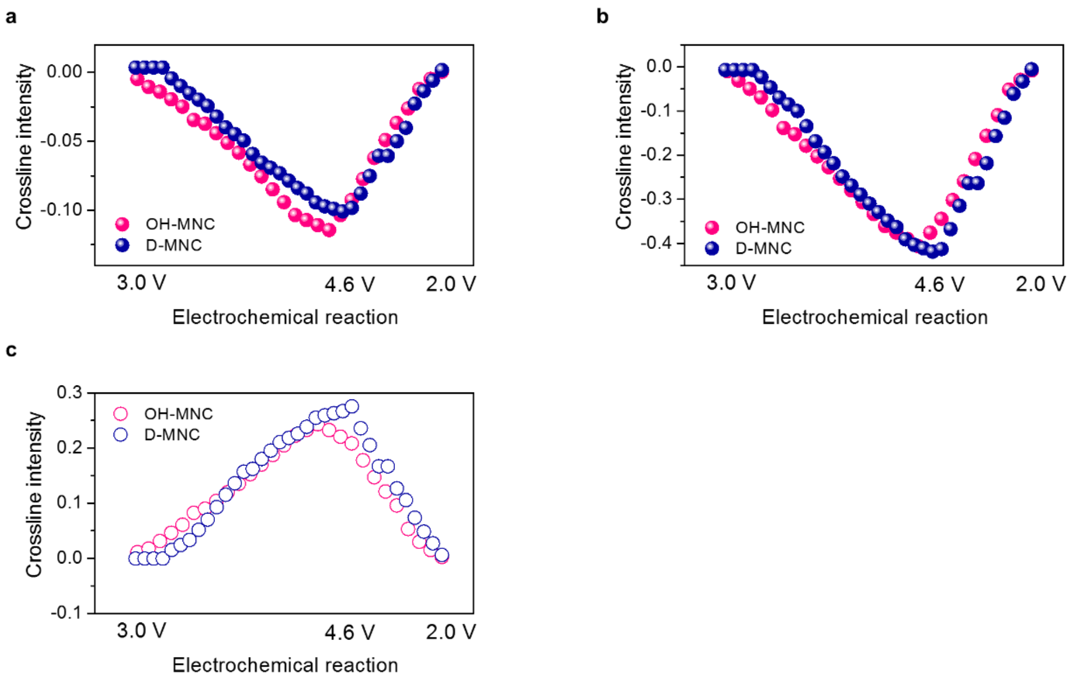

Supplementary Figure 9 | Crossline intensity of Co K-edge *operando* XANES spectra during 10th cycle. Crossline intensity of a) peak A; b) peak B; and c) peak C

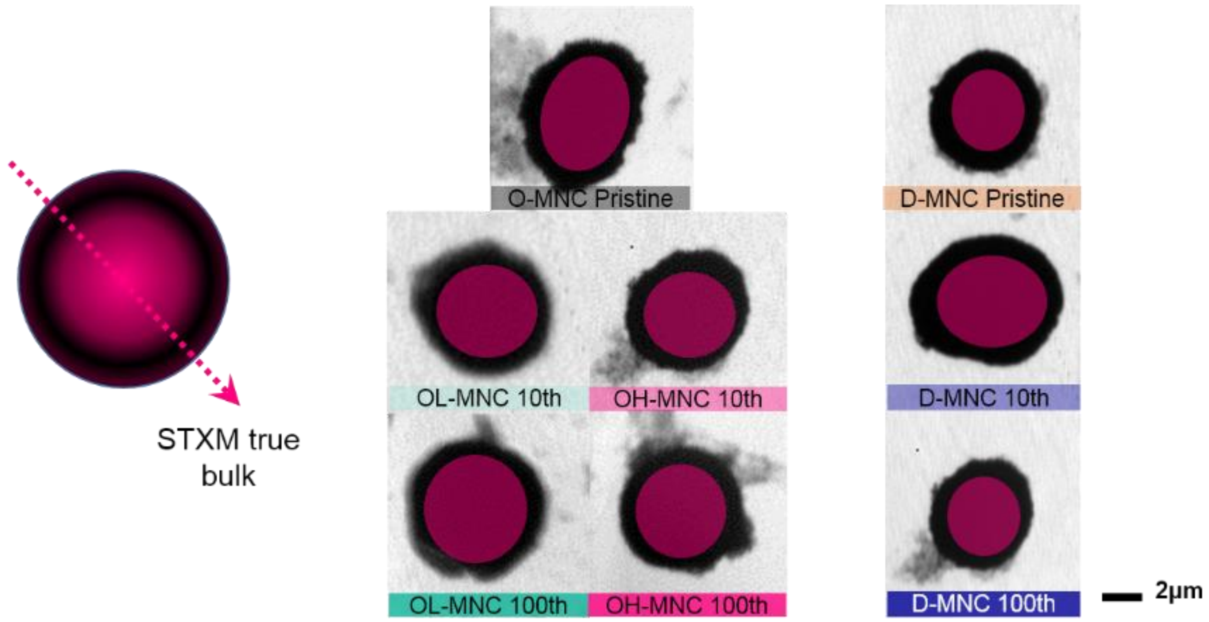

Supplementary Figure 10 | STXM images of pristine O-MNC, D-MNC and cycled samples (10th and 100th cycled and discharged state of OL-MNC, OH-MNC and D-MNC). Colored region indicates the signal area.

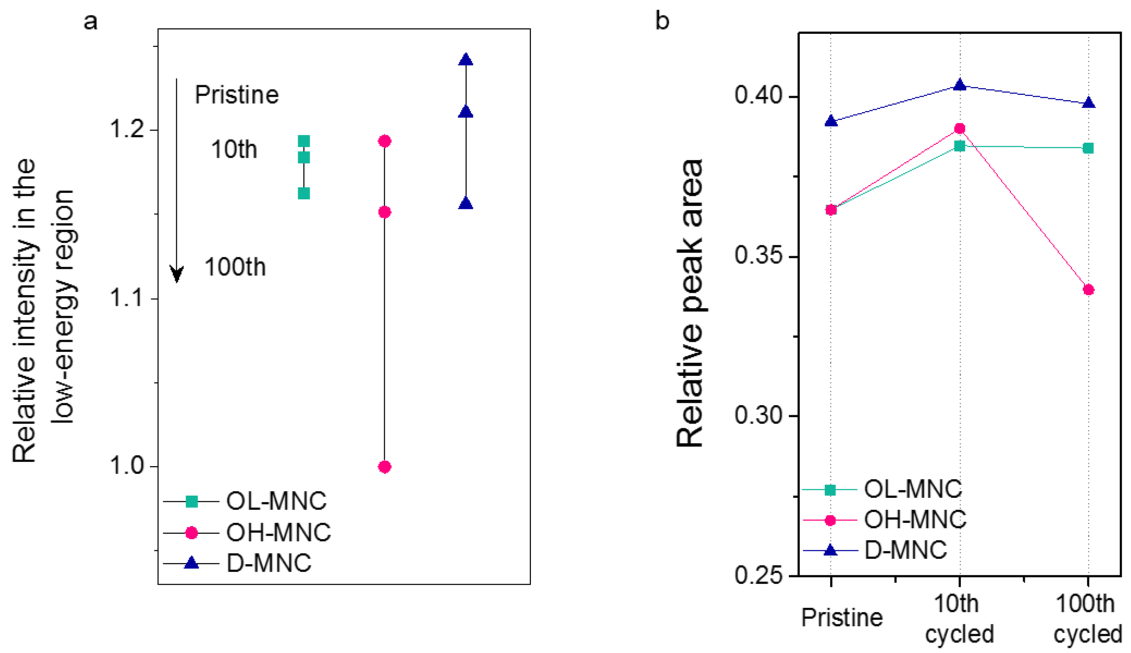

Supplementary Figure 11 | O K-edge SXAS result. Variation of the integrated intensity in the a) pre-edge peak region (shaded region in Fig. 3a-c) and b) ratio of the region below 534 eV (O2*p*–TM3*d*) with respect to the above 534 eV (O2*p*–TM4*sp*) for O K-edge SXAS. After 10th cycle, we expected that the activation process of Li_2TMO_3 phase with oxygen evolutions (oxygens with shared electrons) causes the increase of the hole (increase ratio of 3*d*/4*sp)*. However, OH-MNC shows significant variation and low number of holes compared to D-MNC. After the 100th cycle, the variation of the ratio is proportion to the voltage decay rate of OL-MNC, OH-MNC and D-MNC has shown in Figure.1. From the results, we can expect that significant increase and low number of holes after 10th cycle causes irreversible behavior of redox reaction. Furthermore, Cation migration and atomic rearrangement with decreasing the hole (decrease ratio of 3*d*/4*sp)* occurs to decrease the instability of structure originated from continuous oxygen evolution during cycling

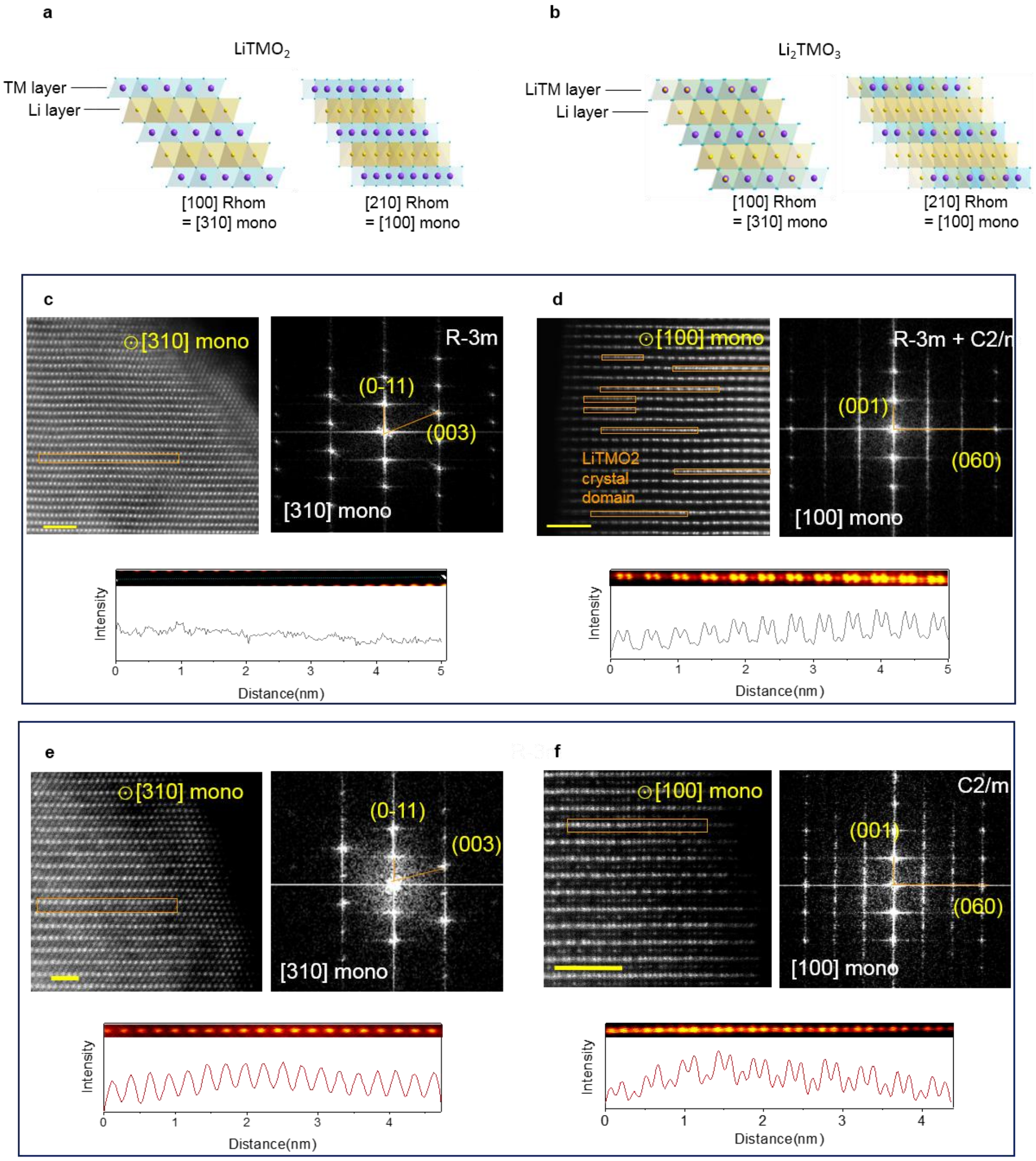

Supplementary Figure 12 | Structure model of $LiTMO_2$ and Li_2TMO_3 phase, which consists of Li-excess 3*d*-transition-metal oxide a) $LiTMO_2$ phase b) Li_2TMO_3 phase along [310] mono and [100] mono zone axis**; HAADF-STEM images, FFT patterns and signal profile of pristine O-MNC and D-MNC samples along [310] mono and [100] mono zone axis.** c) O-MNC [310]mono d) O-MNC [100]mono e) D-MNC [310]mono f) D-MNC [100]mono. Scale bar denote 2 nm.

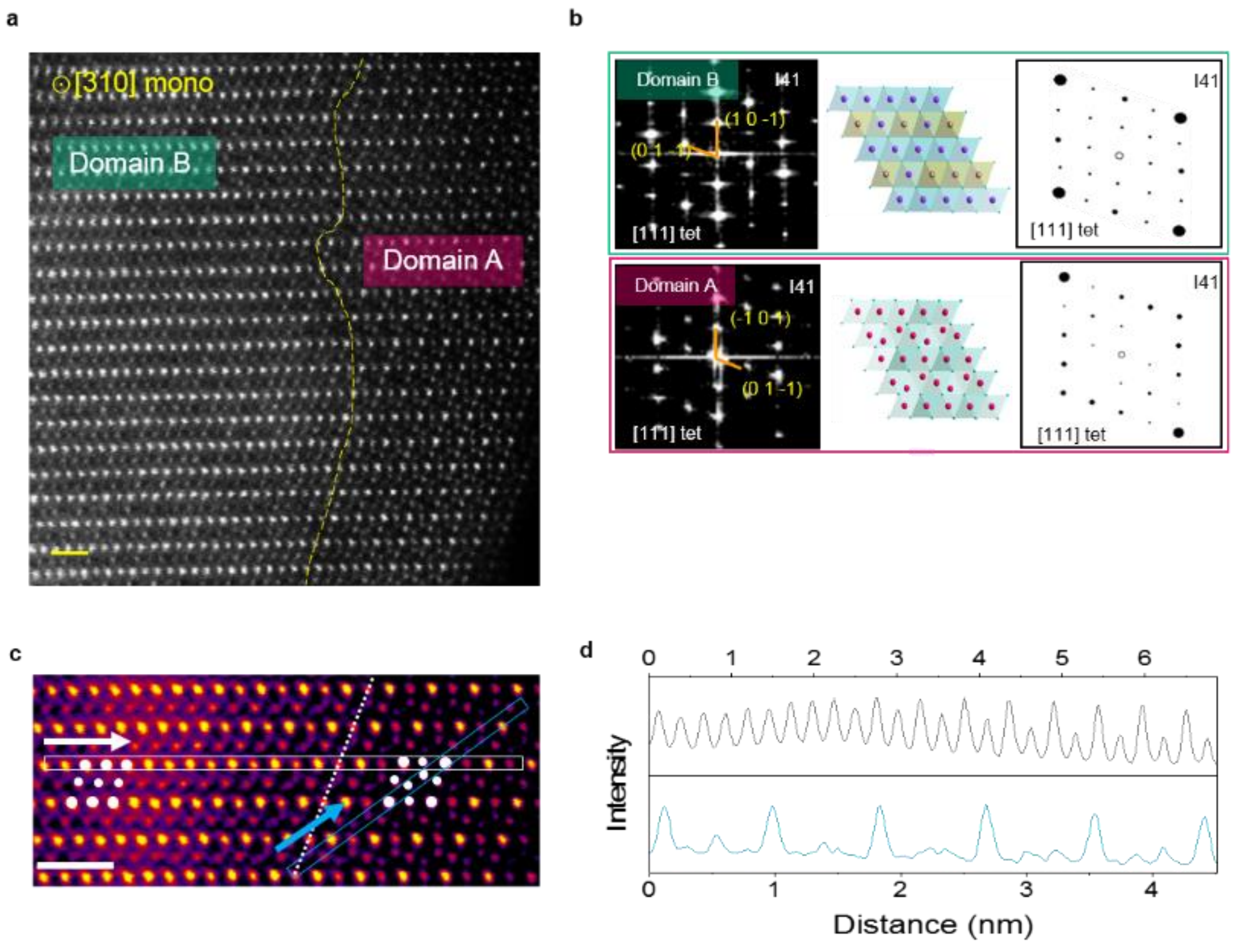

Supplementary Figure 13 | Atomic structure analysis and identifying. a) $[310]_{mono}$ direction HAADF-STEM image of 100th cycled OH-MNC and b) collected FFT patterns were matched with simulated atomic structure model and simulated FFT pattern, respectively. c) Magnified HAADF-STEM image containing domain boundary. d) Signal profile of the region marked in grey and blue in image. In order to clarify the crystal structure of domain A and B, FFT patterns and HAADF-STEM signal profile fitting revealing atomic distance were matched with simulated result by using atomic structure model. Domain A and B collected along $[310]_{mono}$ direction is well matched with simulated FFT patterns of tetragonal structure $LiMn_3O_4$ spinel-like phase and Mn_3O_4 spinel phase with I41 space group along $[111]_{tet}$ direction, respectively.[4-6] Scale bar denote 1 nm.

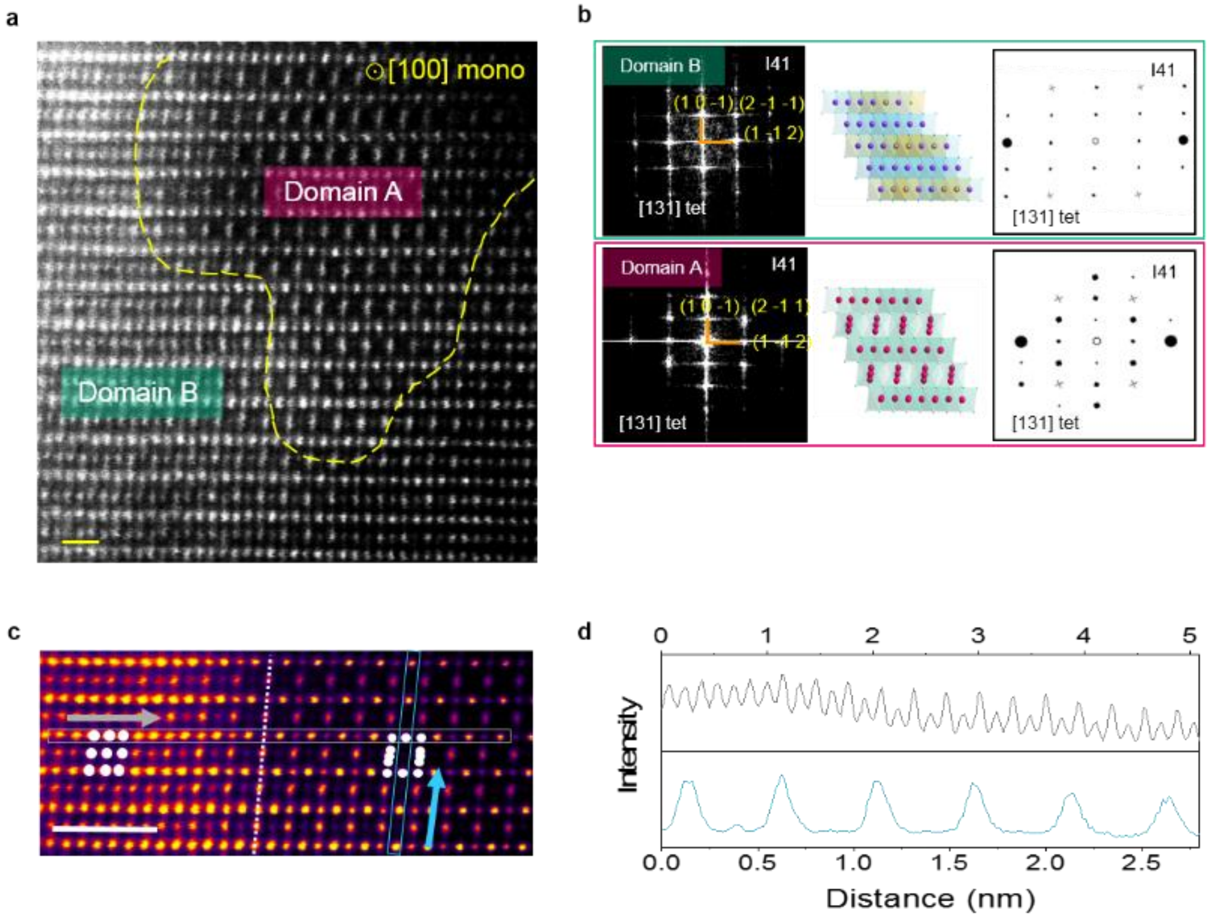

Supplementary Figure 14 | Atomic structure analysis and identifying. a) $[100]_{mono}$ direction HAADF-STEM image of 100th cycled OH-MNC and b) collected FFT patterns were matched with simulated atomic structure model and simulated FFT pattern, respectively. c) Magnified HAADF-STEM image containing domain boundary. d) Signal profile of the region marked in grey and blue in image. Simulated FFT patterns along $[131]_{tet}$ direction of $LiMn_3O_4$ spinel-like phase and Mn_3O_4 spinel phase is also well matched with collected FFT patterns of Domain A and Domain B which collected from HAADF-STEM image along $[100]_{mono}$ direction. HAADF-STEM signal peak of the region marked in grey and blue in supplementary figure also well-matched with atomic distance of structure model. Scale bar denote 1 nm.

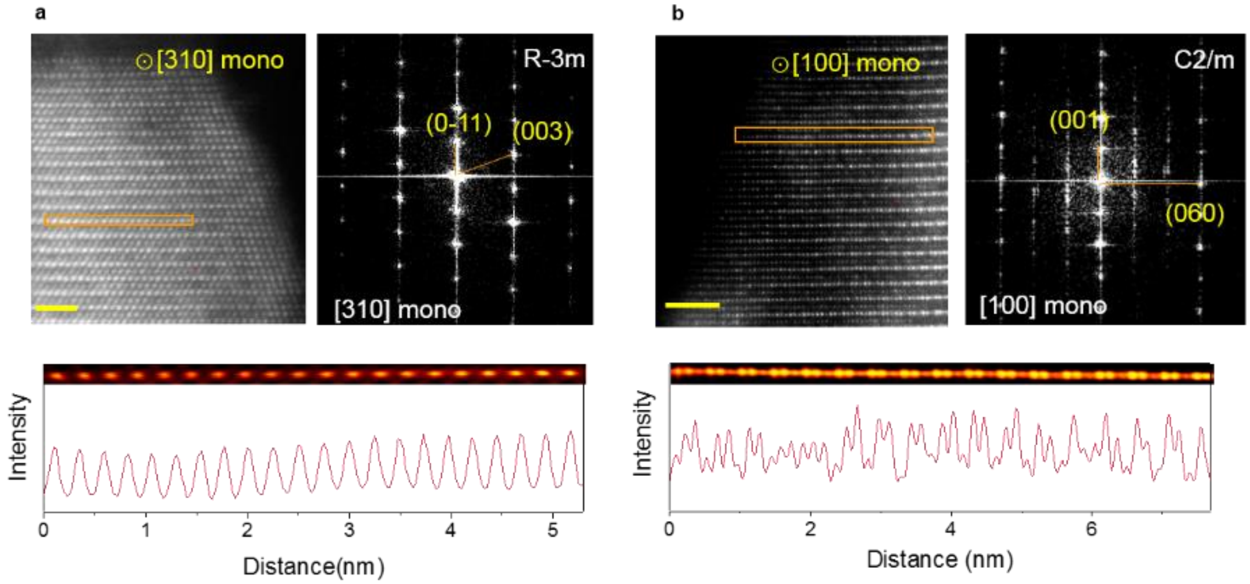

Supplementary Figure 15 | HAADF-STEM images, FFT patterns and signal profile of 100th cycled D-MNC samples along [310]mono and [100]mono zone axis. a) D-MNC [310]mono b) D-MNC [100]mono. Scale bar denote 2 nm.

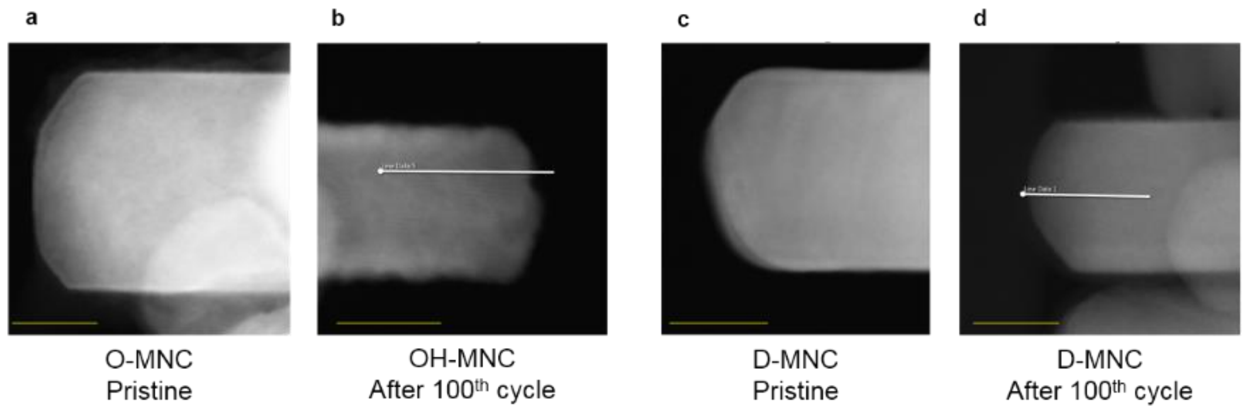

Supplementary Figure 16 | HR-TEM image of pristine O-MNC/D-MNC samples and 100th cycled OH-MNC/D-MNC for EDS mapping analysis. a) Pristine O-MNC b) 100th cycled OH-MNC c) Pristine D-MNC d) 100th cycled D-MNC. Scale bar denote 25nm.

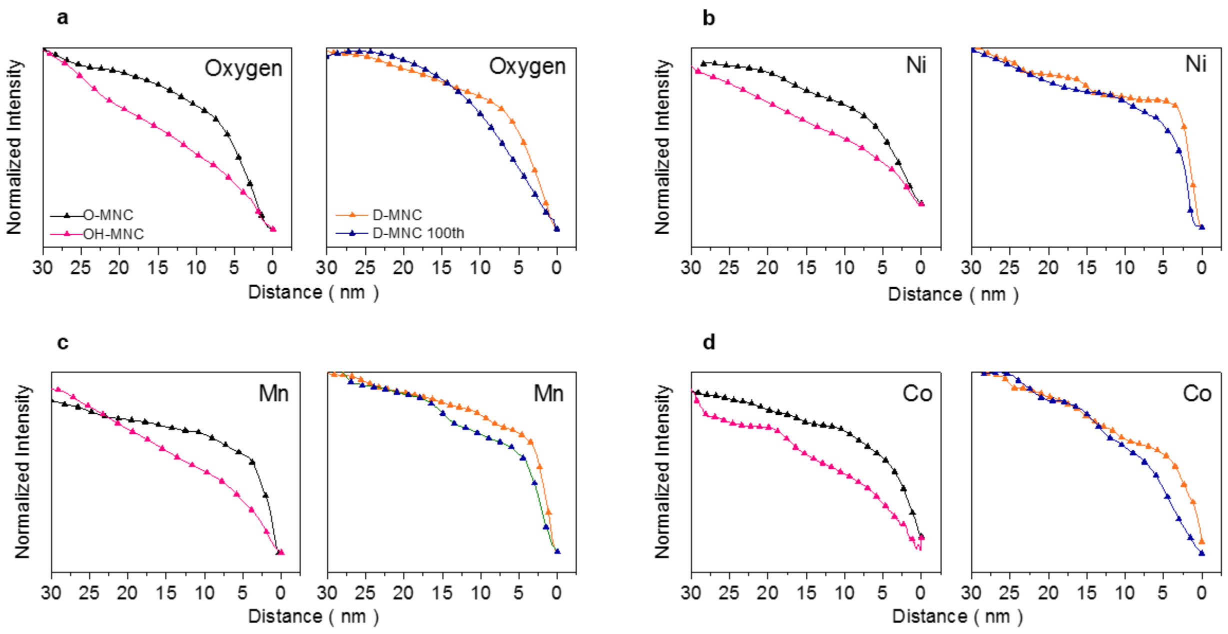

Supplementary Figure 17 | EDS line scan signal counts of Pristine and 100th cycled OH-MNC /D-MNC samples. Degree of elemental signal count change of a) Oxygen, b) Ni, c) Mn, and d) Co from outer-surface to 30nm.

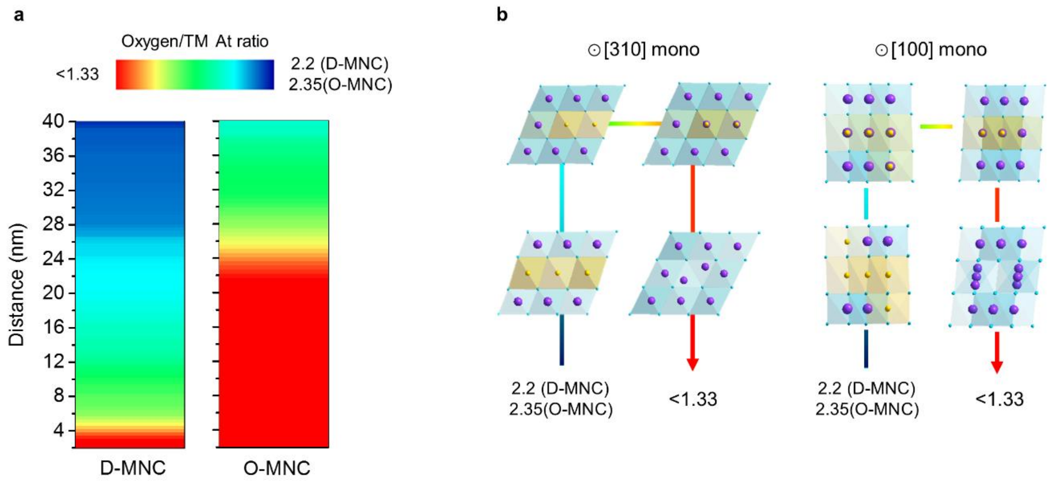

Supplementary Figure 18 | Comparison of surface atomic rearrangement of OH-MNC and D-MNC on long cycling. a) The image plot of oxygen ratio line mapping (30nm long) for cycled OH-MNC and D-MNC from surface region. The intensity is color coded with the scale bare shown on right. b) Schematic of surface structure rearrangement at $[310]_{mono}$ and $[100]_{mono}$ zone direction.

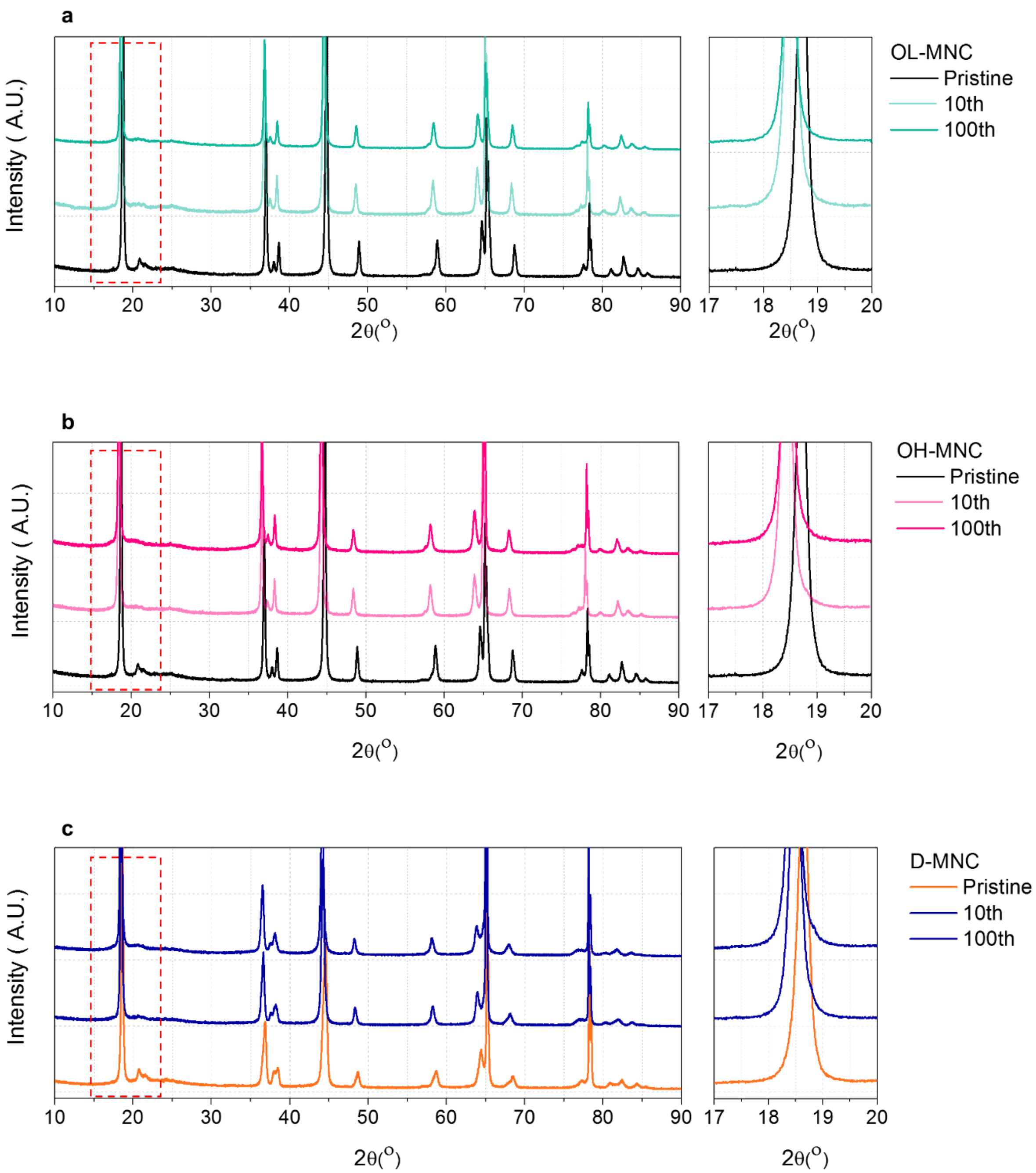

Supplementary Figure 19 | Powder XRD pattern of pristine O-MNC, D-MNC and cycled samples (10 and 100 cycles). a) OL-MNC, b) OH-MNC, and c) D-MNC.

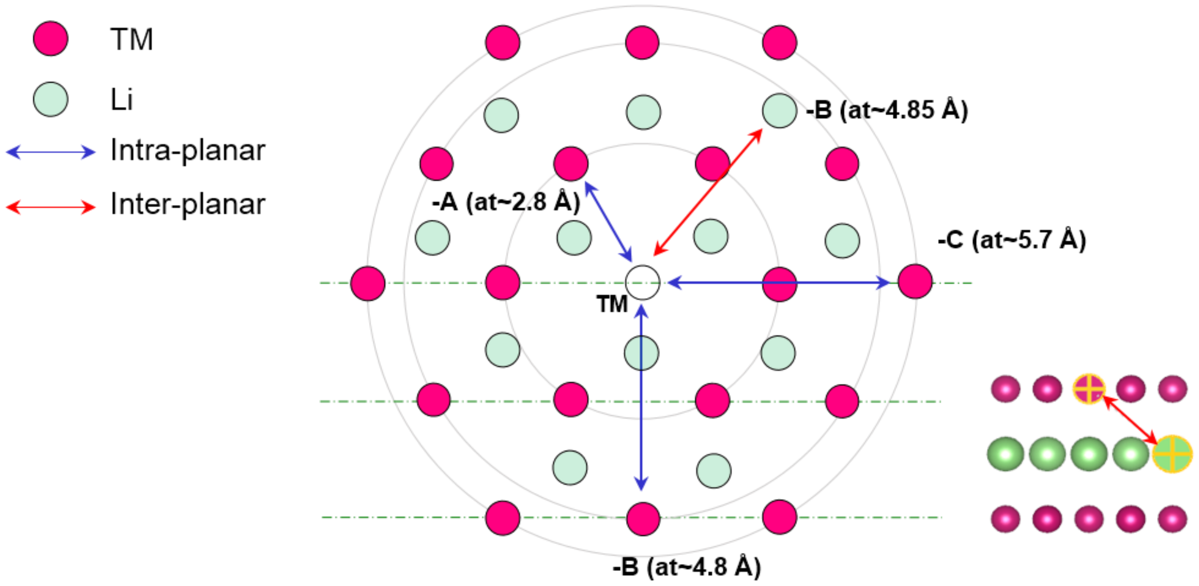

Supplementary Figure 20 | Schematic diagram of scattering paths in the TM atomic arrangements within the ab plane.[7] Schematic diagram shows the possible intra/inter-planar scattering paths in the TM atomic array within the ab plane. The peak at ~ 1.8 Å (TM-O) and ~2.8 Å (TM-A) corresponds to six-coordinated oxygen and TM of the nearest neighboring atom around the TM atom is important to determine the correct structural information, respectively. The peak of ~4.8 Å (TM-B) is mainly contributed from TM around at ~4.8 Å and substituted TM at Li sites located ~ 4.8 Å from TM. The peak of ~5.7 Å (TM-C) is mainly contributed from TM around at ~5.7 Å Moreover, significant variation has been observed in the higher FT peaks above ~4.3 Å which shows useful structural information for Li-excess 3*d*-transition metal oxides.

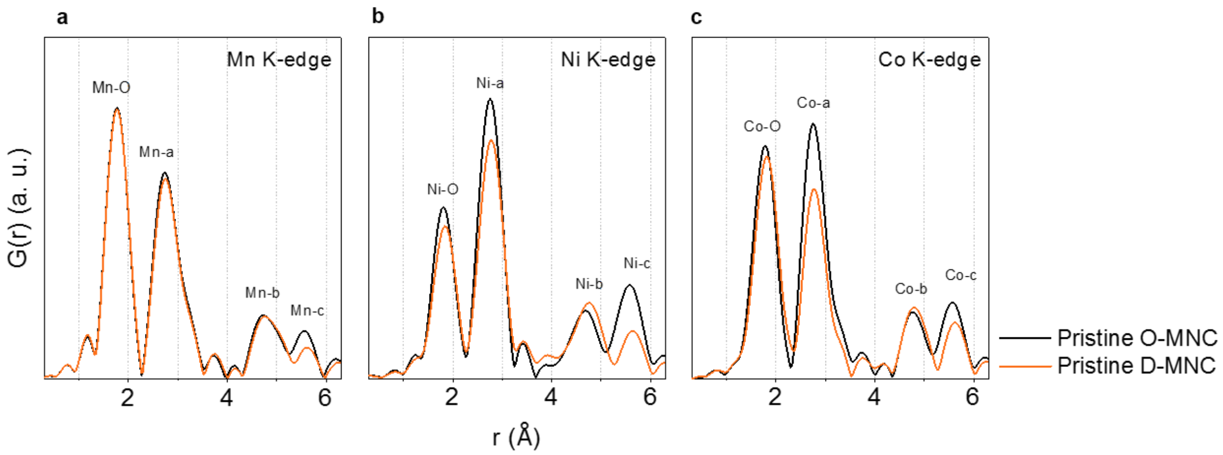

Supplementary Figure 21 | Radial distribution function (RDF) of (a) Mn, (b) Ni, and (c) Co K-edge k3-weighted EXAFS spectra as a function of interatomic distance for pristine electrode of OH-MNC and D-MNC. Pristine O-MNC and D-MNC, show higher Mn-O peak intensity than Mn-A peak, which is typical EXAFS data at the Mn K-edge. This feature originates from the specific structure of Li-excess 3*d*-transition-metal oxide which has Li_2MnO_3 and $LiTMO_2$ domains.[8]

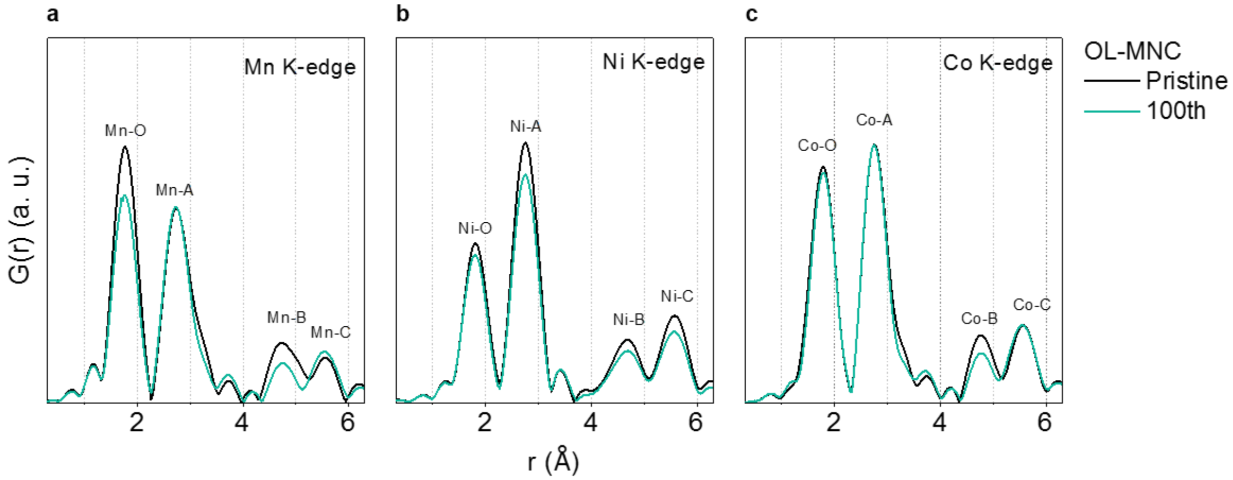

Supplementary Figure 22 | Radial distribution function (RDF) of (a) Mn, (b) Ni, and (c) Co K-edge k3-weighted EXAFS spectra as a function of interatomic distance for 100th cycled electrode of OL-MNC. Radial distribution function (RDF) of (a) Mn, (b) Ni, and (c) Co K-edge k3-weighted EXAFS spectra as a function of interatomic distance for Pristine O-MNC, 10th cycled and 100th cycled electrode of OL-MNC.

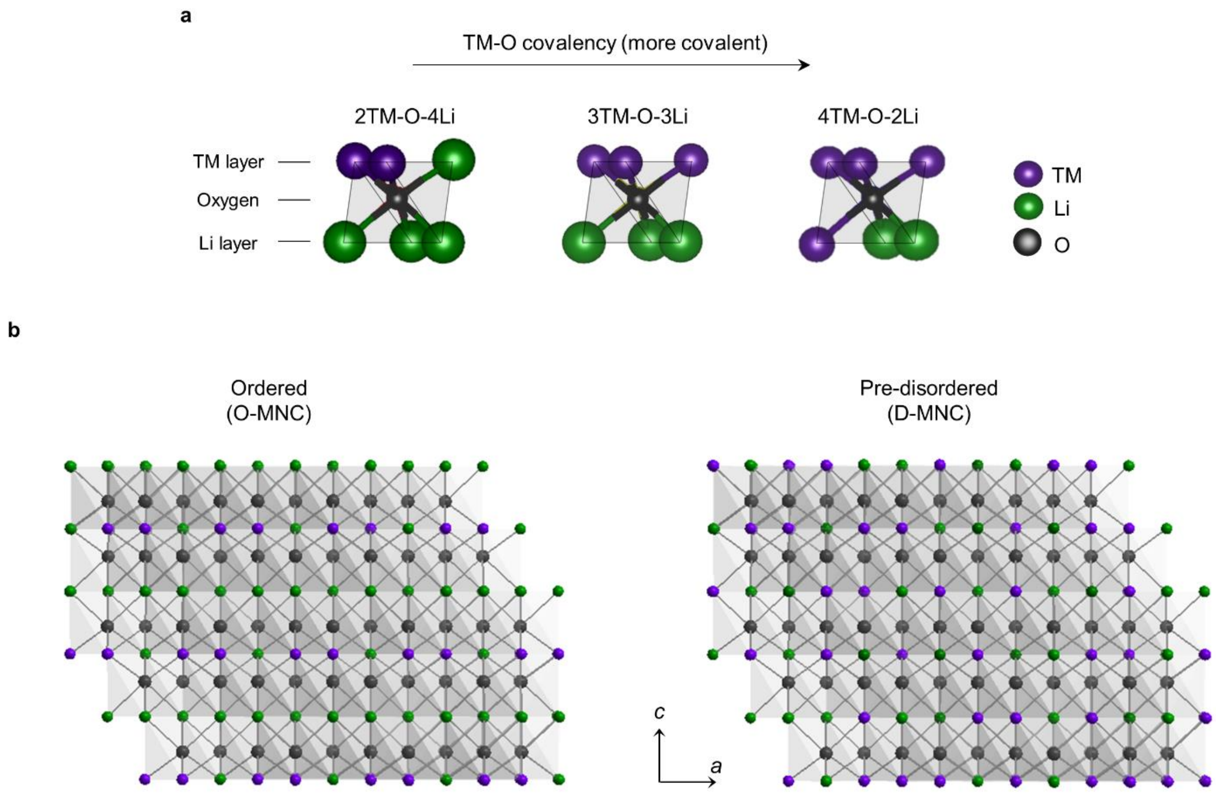

Supplementary Figure 23 | Oxygen-centred structure model of pristine O-MNC and D-MNC (**a**) Three oxygen-centred octahedron (M_6O) of Li-excess material. Each oxygen anions coordinated by six cations with different portion of Li^+ and transition-metal in TM and Li layer. 2TM-O-4Li, 3TM-O-3Li and 4TM-O-2Li octahedron represent the base structure of Li_2TMO_3, $LiTMO_2$ and cation-disordered-$LiTMO_2$ (**b**) Oxygen-centred macroscopic structure model of pristine O-MNC and D-MNC along $[100]_{mono}$.

Supplementary Table 1 | Cell parameters for O-MNC and D-MNC.

		O-MNC	D-MNC
R-3m phase	a- value (Å)	2.8517	2.8714
	c- value (Å)	14.2356	14.2611
C2/m phase	a- value (Å)	4.9433	4.9561
	b- value (Å)	8.5571	8.5667
	c- value (Å)	5.0371	5.0364

Supplementary Table 2 | Structure parameters for O-MNC and D-MNC.

Sample	Element	*x*	*y*	*z*	Occupancy
O-MNC	Li(1)	0.000000	0.000000	0.000000	0.970
	O(1)	0.000000	0.000000	0.24159(6)	1
	Co(1)	0.000000	0.000000	0.500000	0.163
	Ni(1)	0.000000	0.000000	0.500000	0.1474
	Mn(1)	0.000000	0.000000	0.500000	0.510
	Li(2)	0.000000	0.000000	0.500000	0.180
	Co(2)	0.000000	0.000000	0.000000	0.007
	Ni(2)	0.000000	0.000000	0.000000	0.0226
**Sample**	**Element**	***x***	***y***	***z***	**Occupancy**
D-MNC	Li(1)	0.000000	0.000000	0.000000	0.9017
	O(1)	0.000000	0.000000	0.24134(6)	1.000
	Co(!)	0.000000	0.000000	0.500000	0.033
	Ni(1)	0.000000	0.000000	0.500000	0.2217
	Mn(1)	0.000000	0.000000	0.500000	0.550
	Li(2)	0.000000	0.000000	0.500000	0.1953
	Co(2)	0.000000	0.000000	0.000000	0.010
	Ni(2)	0.000000	0.000000	0.000000	0.0983

Supplementary Table 3 | Electrochemical performance result value of the OL-MNC, OH-MNC and D-MNC.

Sample	0.1C-rate Discharge capacity (mAh g^{-1}) 2.0 – 4.6 V (versus. Li-metal) 1C=200mA g^{-1}	Coulumbic efficiency (%)	1.0 C-rate Discharge capacity (mAh g^{-1})	Cycle retention (%)
OL-MNC	256	90	150 (1C = 256mA g^{-1})	93.5%
OH-MNC	256	90	200 (1C = 256mA g^{-1})	93.8%
D-MNC	250	93	200 (1C = 250mA g^{-1})	93.3%

Supplementary Table 4 | Average discharge voltage values of the OL-MNC, OH-MNC and D-MNC at each cycle.

Sample	Average voltage (V)					Voltage decay (V)
	10cyc	25cyc	50cyc	75cyc	100cyc	V(100)-V(10)
OL-MNC	3.722	3.718	3.707	3.696	3.690	0.0316
OH-MNC	3.599	3.513	3.422	3.335	3.282	0.317
D-MNC	3.694	3.668	3.655	3.647	3.637	0.056

Supplementary Table 5 | XANES peak energy values of the OL-MNC, OH-MNC and D-MNC at Initial, charged and discharged state.

OL-MNC 10th	Initial state (eV)	ΔOx	Charged state (eV)	ΔRed	Discharged state (eV)	References (eV)
Mn K-edge	6558.89 (3.30+)	0.23	6559.89 (3.53+)	0.23	6558.89 (3.30+)	6558.00 (Mn_2O_3) 6561.00 (MnO_2)
Ni K-edge	8349.15 (2.23+)	0.61	8352.21 (2.84+)	0.61	8349.15 (2.23+)	8348.00 (NiO) 8353.00 ($LiNiO_2$) 8355.50 (NiO_2)
Co K-edge	7727.38 (2.47+)	0.38	7728.90 (2.85+)	0.38	7727.38 (2.47+)	7725.50 (CoO) 7729.50 ($LiCoO_2$)

OH-MNC 10th	Initial state (eV)	ΔOx	Charged state (eV)	ΔRed	Discharged state (eV)	References (eV)
Mn K-edge	6558.39 (3.19+)	0.46	6560.40 (3.65+)	0.35	6558.89 (3.30+)	6558.00 (Mn_2O_3) 6561.00 (MnO_2)
Ni K-edge	8349.68 (2.34+)	0.73	8353.21 (3.08+)	0.73	8349.68 (2.34+)	8348.00 (NiO) 8353.00 ($LiNiO_2$) 8355.50 (NiO_2)
Co K-edge	7727.36 (2.47+)	0.51	7729.40 (2.98+)	0.51	7727.36 (2.47+)	7725.50 (CoO) 7729.50 ($LiCoO_2$)
**D-MNC 10th**	**Initial state (eV)**	**ΔOx**	**Charged state (eV)**	**ΔRed**	**Discharged state (eV)**	**References (eV)**
Mn K-edge	6558.39 (3.19+)	0.34	6559.89 (3.53+)	0.29	6558.64 (3.24+)	6558.00 (Mn_2O_3) 6561.00 (MnO_2)
Ni K-edge	8348.85 (2.17+)	0.71	8352.38 (2.88+)	0.71	8348.85 (2.17+)	8348.00 (NiO) 8353.00 ($LiNiO_2$) 8355.50 (NiO_2)
Co K-edge	7726.85 (2.34+)	0.51	7728.90 (2.85+)	0.51	7726.85 (2.34+)	7725.50 (CoO) 7729.50 ($LiCoO_2$)

OH-MNC 100th	Initial state (eV)	ΔOx	Charged state (eV)	ΔRed	Discharged state (eV)	References (eV)
Mn K-edge	6558.89 (3.30+)	0.35	6560.40 (3.65+)	0.35	6558.89 (3.30+)	6558.00 (Mn_2O_3) 6561.00 (MnO_2)
Ni K-edge	8350.16 (2.33+)	0.75	8353.21 (3.08+)	0.75	8350.16 (2.33+)	8348.00 (NiO) 8353.00 ($LiNiO_2$) 8355.50 (NiO_2)
Co K-edge	7727.36 (2.47+)	0.38	7728.91 (2.85+)	0.38	7727.36 (2.47+)	7725.50 (CoO) 7729.50 ($LiCoO_2$)
**D-MNC 100th**	**Initial state (eV)**	**ΔOx**	**Charged state (eV)**	**ΔRed**	**Discharged state (eV)**	**References (eV)**
Mn K-edge	6558.38 (3.18+)	0.37	6559.94 (3.55+)	0.37	6558.38 (3.18+)	6558.00 (Mn_2O_3) 6561.00 (MnO_2)
Ni K-edge	8349.15 (2.23+)	0.85	8353.19 (3.08+)	0.85	8349.15 (2.23+)	8348.00 (NiO) 8353.00 ($LiNiO_2$) 8355.50 (NiO_2)
Co K-edge	7726.85 (2.34+)	0.51	7728.90 (2.85+)	0.51	7726.85 (2.34+)	7725.50 (CoO) 7729.50 ($LiCoO_2$)

Supplementary Table 6 | Theoretical and experimental O/TM ratio result of pristine O-MNC/D-MNC and 100th cycled OH-MNC/D-MNC from EDS mapping analysis.

Sample	**O-MNC Pristine**<br>$Li_{1.15}Mn_{0.51}Ni_{0.17}Co_{0.17}O_2$<br>$0.35(Li_2MnO_3)\text{-}0.65(LiMn_{0.38}Ni_{0.31}Co_{0.31}O_2)$	**D-MNC Pristine**<br>$Li_{1.09}Mn_{0.550}Ni_{0.320}Co_{0.043}O_2$<br>$0.2(Li_2MnO_3)\text{-}0.8(LiMn_{0.5}Ni_{0.44}Co_{0.063}O_2)$
**Theoretical value**	2.350	2.200
**Experimental value**	2.238	2.145
**correction factor**	1.050	1.025
Sample	**OH-MNC**<br>after 100th cycle	**D-MNC**<br>after 100th cycle
**Experimental value**	2.0156	2.102
**Theoretical value**	2.116	2.156

Supplementary Note 1. Rietveld refinement analysis

As the Li-excess 3*d*-transition metal oxide is the mixture Li_2MnO_3 and $LiMO_2$ (M=Mn, Ni and Co) components, the Rietveld refined XRD patterns have been analyzed based on Li_2MnO_3 ($C2/m$) and $LiMn_{1/3}Ni_{1/3}Co_{1/3}O_2$ ($R\bar{3}m$) for both O-MNC and D-MNC. From the refinement result, the cell parameters of O-MNC and D-MNC based on $R\bar{3}m$ space group are (a=2.8517, c=14.2356) and (a=2.8714, c=14.2611). Furthermore, a structure parameter representing the Li and TM atomic occupancy shows the degree of cation disordering between TM and Li layer. The results indicate that more Ni and Co ions have occupied the Li site in D-MNC (total 0.10mol) compared to O-MNC (total 0.03mol). The different stoichiometry of Ni and Co ratio affect the structure of material due to the different octahedral site stabilization energy of TM ion (OSSE- Ni^{3+} : -12.67*Dq* / Co^{3+} : -21.33*Dq*).[9] A larger OSSE value for the Co^{3+} ions makes the TM migration difficult. Therefore, D-MNC with High Ni content and Low Co content shows more disorder structure compared to O-MNC with high Co content.

Supplementary Note 2. EDS spectrometer analysis

High efficient EDS spectrometer analysis were conducted to reveal the correlation between phase transition and oxygen deficiency. Comparing with the O/TM atomic ratio of pristine O-MNC and D-MNC according to the area mapping, the average ratio of pristine O-MNC and D-MNC are 2.238 and 2.145. These experimental values are remarkably similar to theoretical values calculated from O-MNC = 0.35(Li_2MnO_3)-0.65($LiMn_{0.38}Ni_{0.31}Co_{0.31}O_2$) and D-MNC = 0.2($Li_2MnO_3$)-0.8($LiMn_{0.5}Ni_{0.44}Co_{0.063}O_2$) are 2.35 and 2.2, respectively. From this result, we deducted correction factors and calculated correction O/TM ratio of cycled OH-MNC: 2.11647 and D-MNC: 2.155897 (Supplementary Table 6). Associating the normalized oxygen line mapping intensity data in supplementary figure with corrected O/TM ratio, the oxygen deficiency in accordance with the distance from outer-surface of 100th cycled OH-MNC and D-MNC were demonstrated in color maps (Supplementary Fig. 18a). In contrast to D-MNC, O/TM ratio below 1.33 were detected from outer-surface to 23nm in OH-MNC which suffered severe structural transition. Oxygen loss arose from redox mechanism and band structure of Li-excess 3*d*-transition-metal oxide leads cation disordering, which is accelerated at outer-surface directly reacted with electrolyte. Phase transition sequence from well-ordered layered to TM_3O_4 spinel phase with I41 space group observed in both $[310]_{mono}$ and $[100]_{mono}$ direction has direct correlation with oxygen deficiency (Supplementary Fig. 18b).

Supplementary References

1. Simonin L, Colin JF, Ranieri V, Canévet E, Martin JF, Bourbon C, *et al.* In situ investigations of a Li-rich Mn-Ni layered oxide for Li-ion batteries. *J. Mater. Chem.* **22**, 11316–11322 (2012).

2. Wang J, Zhou J, Hu Y, Regier T. Chemical interaction and imaging of single Co_3O_4/graphene sheets studied by scanning transmission X-ray microscopy and X-ray absorption spectroscopy. *Energy Environ. Sci.* **6**, 926–934 (2013).

3. Kiss AM, Harris WM, Wang S, Vila-Comamala J, Deriy A, Chiu WKS. In-situ observation of nickel oxidation using synchrotron based full-field transmission X-ray microscopy. *Appl. Phys. Lett.* **102**, 053902 (2013).

4. Shimoda K, Oishi M, Matsunaga T, Murakami M, Yamanaka K, Arai H, *et al.* Direct observation of layered-to-spinel phase transformation in Li_2MnO_3 and the spinel structure stabilised after the activation process. *J. Mater. Chem. A* **5**, 6695–6707 (2017).

5. Yan P, Nie A, Zheng J, Zhou Y, Lu D, Zhang X, *et al.* Evolution of lattice structure and chemical composition of the surface reconstruction layer in $Li_{1.2}Ni_{0.2}Mn_{0.6}O_2$ cathode material for lithium ion batteries. *Nano Lett.* **15**, 514–522 (2015).

6. Yan P, Xiao L, Zheng J, Zhou Y, He Y, Zu X, *et al.* Probing the Degradation Mechanism of Li_2MnO_3 Cathode for Li-Ion Batteries. *Chem. Mater.* **27**, 975–982 (2015).

7. Kim MG, Yo CH. X-ray Absorption Spectroscopic Study of Chemically and Electrochemically Li Ion Extracted $Li_yCo_{0.85}Al_{0.15}O_2$ Compounds. *J. Phys. Chem. B* **103**, 6457–6465 (1999).

8. Rana J, Kloepsch R, Li J, Stan M, Schumacher G, Winter M, *et al.* Structural Changes in a Li-Rich $0.5Li_2MnO_3 \bullet 0.5LiMn_{0.4}Ni_{0.4}Co_{0.2}O_2$ Cathode Material for Li-Ion Batteries: A Local Perspective. *J. Electrochem. Soc.* **163**, A811–A820 (2016).

9. Choi S, Manthiram A. Factors influencing the layered to spinel-like phase transition in layered oxide cathodes. *J. Electrochem. Soc.* **149**, A1157–A1163 (2002).
